# Primary splenic Alveolar Echinococcosis: a case report from Germany

**DOI:** 10.1007/s15010-026-02911-x

**Published:** 2026-08-03

**Authors:** Guenter Froeschl, Thomas Vogel, Leon Gehri, Thomas F. E. Barth, Hanspeter Frey, Claudia Wallrauch, Camilla Rothe

**Affiliations:** 1 Institute of Infectious Diseases and Tropical Medicine, University Hospital, LMU Munich, Munich, Germany; 2 Department of General- and Visceral Surgery, Hospital of Starnberg, Starnberg, Germany; 3 Institute of Pathology, University of Ulm, Ulm, Germany

**Keywords:** Echinococcus multilocularis, Alveolar Echinococcosis, Spleen, Extra-hepatic, Cystic abdominal lesion Germany

## Abstract

**Purpose:**

We are reporting on a rare case of a primary manifestation of Alveolar Echinococcosis in the spleen in a German patient.

**Methods:**

The patient was initially presented to her gastroenterologist and subsequently referred to a district hospital for surgical management. The further antiparasitic treatment was taken over by the Institute of Infectious Diseases and Tropical Medicine, LMU University Hospital Munich, Germany.

**Case report:**

A 59-year-old German woman presented to her gastroenterologist with weight loss and abdominal discomfort. An ultrasonographic finding of a splenic lesion was further evaluated by abdominal CT and MRI, interpreted initially as a hamartoma by the radiologists. Following partial splenectomy, where an accidental intra-operative cyst rupture occured, pathological examination raised suspicion of echinococcosis. An immune-histological analysis of the resected splenic tissue by the German Reference Laboratory for Echinococcosis confirmed Alveolar Echinococcosis. No additional lesions suggestive of echinococcosis were identified. The patient is currently successfully treated with continuous antiparasitic therapy with albendazole.

**Conclusions:**

Primary manifestations of Alveolar Echinococcosis in the spleen are rare, and may be misleading towards other differential diagnoses.

## Introduction

Alveolar Echinococcosis (AE) is a rare anthropozoonotic parasitic infection caused by the larval stage of the tapeworm *Echinococcus multilocularis*. The disease occurs exclusively in the Northern Hemisphere, and foci with higher prevalence are found in Central Europe (Germany, Austria, Switzerland, France) and Asia (China, Siberia) [1]. In Germany, AE is a notifiable infectious disease. For the year 2024, the German Robert-Koch-Institute reported 189 cases of echinococcosis of which 52 were attributed to *E. multilocularis*, the causative agent of AE [2].

The natural life-cycle involves foxes - and to a lesser extent, dogs and cats - as definitive hosts. Eggs shed in the faeces of the definite host contaminate the environment, small rodents serve as intermediate hosts by ingesting eggs; the hatching oncospheres migrate via the portal circulation to the liver, where multilocular cystic lesions develop. After ingesting infected rodents, protoscoleces mature into adult tapeworms in the definitive host’s intestines [1, 3]. Humans become accidental dead-end intermediate hosts through faecal-oral ingestion of eggs via direct contact with infected animals or through contaminated soil or plants. Larval development in humans is slow, often allowing the disease to remain asymptomatic for years or even decades. Unlike Cystic Echinococcosis (CE), AE lesions are not encapsulated and grow in an infiltrative pattern often resembling malignant tumours. In the vast majority of human cases, the liver is the primary site of infection However, metacestodes can relocate via the venous or lymphatic system to various tissues, such as lung, brain, skin or the spleen, where secondary lesions can develop [1, 3]. In symptomatic untreated cases, the 10-year survival rate has historically been reported as below 10%, but has improved significantly with the introduction of albendazole therapy [3]. Incidental discovery of hepatic lesions during routine abdominal ultrasound or cross-sectional imaging is common. However, the heterogeneous radiologic appearance of AE is far less pathognomonic than in CE - including solid, cystic, and mixed lesions - so those lesions are frequently misdiagnosed and delays in appropriate management are frequent. Diagnostic uncertainty is even greater when the primary lesion is extrahepatic, which is distinctly uncommon. We describe a case of a German patient with an incidentally detected primary splenic lesion in whom immune-histological evaluation ultimately confirmed splenic AE.

### Methods

This case report describes the clinical course of a patient with a rare presentation of primary splenic AE, who was managed sequentially by her gastroenterologist, a district hospital, and finally the infectious disease outpatients department of a university hospital. Ethical approval for the publication of this case was obtained from the Ethics Committee of the Medical Faculty of the Ludwig-Maximilians-Universität Munich, Germany (Opinion No. 25-1028-CR). The patient signed an informed consent for publication.

### Case report

**Fig. 1:**
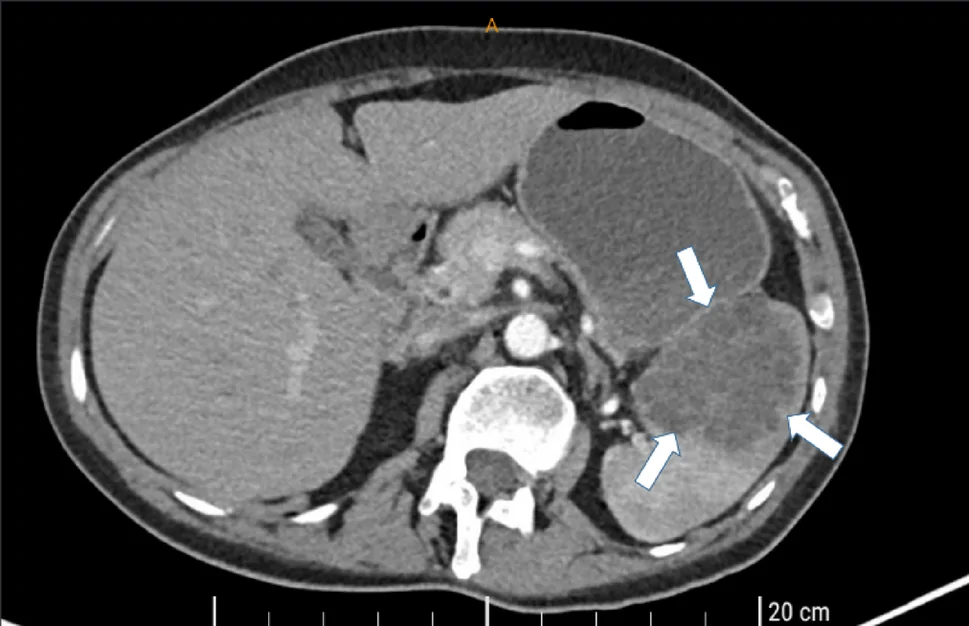
CT scan of the abdomen with a splenic lesion. The CT scan revealed a heterogeneous solid-cystic lesion of the upper pole of the spleen (arrows).

A 59-year old female residing in Upper Bavaria in the South of Germany presented to her gastroenterologist in early 2025 with abdominal discomfort, weight loss, chronic pain and fatigue. Her medical history included rheumatoid arthritis and fibromyalgia. The anamnesis revealed regular leisure activity in the forest including berry picking (even though confirming that no berries from ground level were picked), she has no domestic animals and did not report other close contacts to animals. Abdominal ultrasound revealed a heterogeneous solid-cystic lesion arising from the upper pole of the spleen. Subsequent CT and MRI scans confirmed an 82 × 82 × 54 mm splenic mass (Fig. 1).

**Fig. 2:**
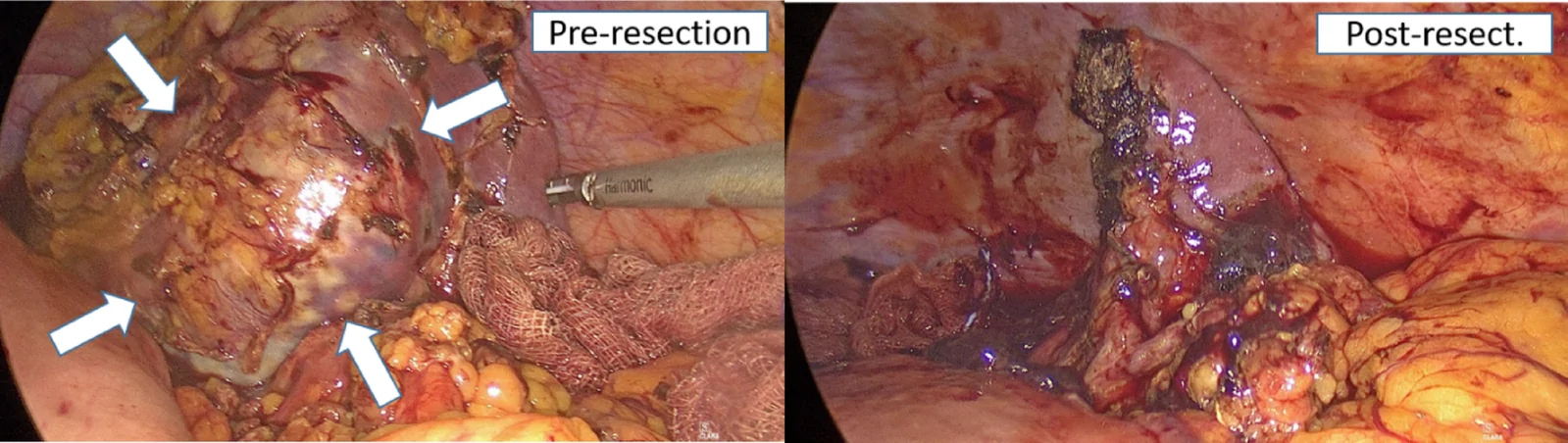
Intra-operative image of a splenic lesion. The spleen with the macroscopically visible mass (arrows) prior to resection (left) and after partial resection of the spleen (right)

No additional suspicious lesions were identified. A simple anechoic hepatic cyst of 13 × 11 × 7 mm was located in the liver segment 2. A hamartoma was at first suspected as aetiology of the splenic lesion, but slight growth in size of the lesion finally resulted in an elective partial resection of the spleen at a local hospital on 09 September 2025 (Fig. 2). During surgical intervention, one cyst ruptured, the site was rinsed and aspirated with saline solution.

**Fig. 3:**
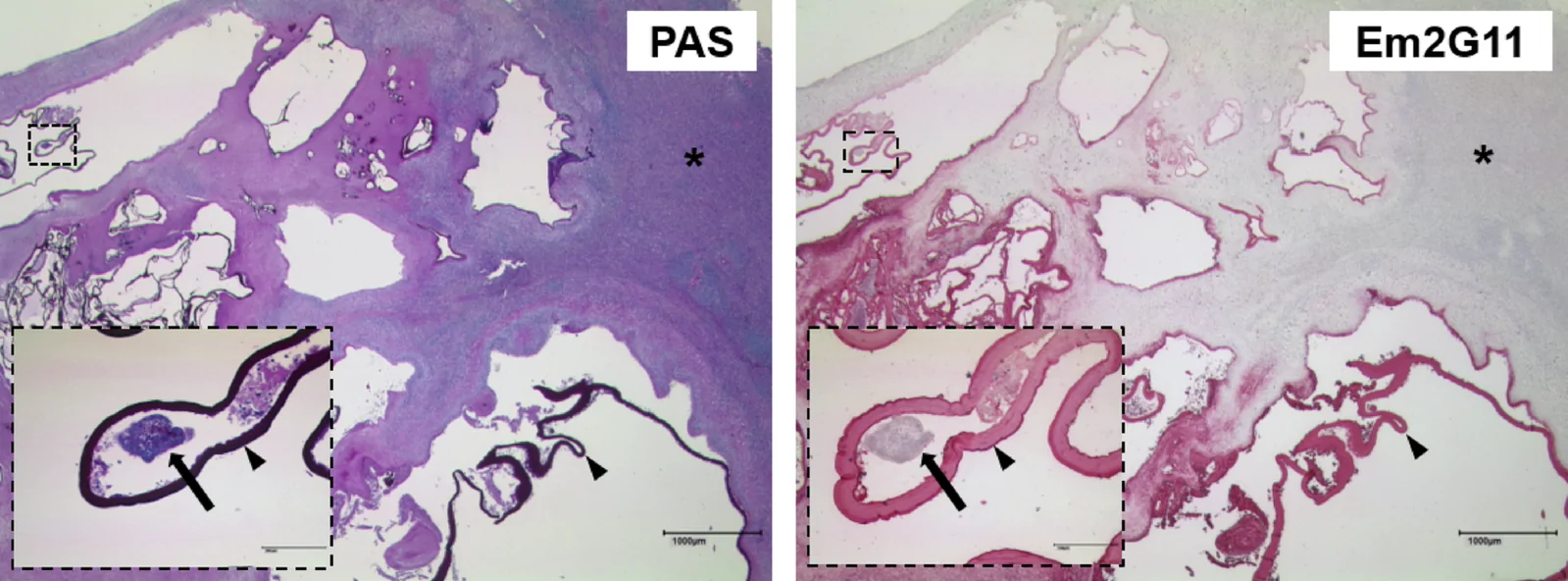
PAS and immuno-histological staining of a splenic *E. multilocularis* lesion. Microscopy of the spleen: PAS-Reaction (left) and Immunohistochemistry (IHC) with the monoclonal antibody Em2G11 (right). The PAS-reaction highlights the parasitic laminated layer (LL, arrowheads) in a profound violet colour. The Em2G11-IHC results in exclusive staining of this structure, confirming *E. multilocularis* infection. The LL is infiltrating the splenic parenchyma (asterisks) in a tubular fashion. Protoscoleces are visible inside the metacestode vesicles (insert, arrows)

The resected material (90 × 90 × 80 mm, 171 g) was sent for pathological evaluation The microscopic investigation of tissue sections showed red and white pulp spleen tissue, surrounding an agglomerate of cystic, partially confluent elements, with histiocytic peripheral infiltration, and PAS (Periodic Acid-Schiff) positive laminated layers. In addition, protoscoleces could be identified. These findings raised suspicion for echinococcal disease. A paraffin block of the resected lesion was forwarded to the German reference laboratory for echinococcosis at the University of Ulm, where an immuno-histological staining using monoclonal antibody Em2G11 - specifically binding to the laminated layer of *E. multilocularis* larvae - showed strong positivity, confirming AE (Fig. 3).

The post-interventional recovery was uncomplicated, and the patient was referred to the Institute of Infectious Diseases and Tropical Medicine of the LMU University Hospital in Munich, Germany, where she presented on 17 September 2025. The post-interventional ultrasound investigation revealed the site of the partial splenectomy in state of re-organization, there was a minor pleural effusion of 2 mm on the left side, and in the left lobe of the liver there was the previously described non-echogenic and non-encapsulated cyst of 11 × 10 × 9 mm.

Serology performed postoperatively demonstrated positive *Echinococcus* IgG ELISA, positive Em2 + serology, and a positive *Echinococcus* immunoblot (bands p7 and p26–28).

In order to rule out additional lesions, an X-ray investigation of the thorax was performed, without pathological findings. Since the patient mentioned recurrent headaches, a CT scan of the brain was performed. No relevant pathological findings were reported either.

Albendazole therapy was initiated at 400 mg twice daily, and is planned for a minimum of two years in accordance with national recommendations [1]. Follow-up visits are scheduled every three months to monitor for new lesions (ultrasound), evolution of serological markers, medication tolerance, and albendazole blood levels (4-hour post-dose).

Except a very mild hair-loss, the patient did not report any side-effects of the albendazole therapy so far (such as headache, dizziness, gastro-intestinal symptoms). Liver function tests and full blood count, which were altered immediately after the surgical intervention, remained at a moderate level of anaemia (pre-interventional haemoglobin 13.6 g/dl; immediately post-intervention 10.3 g/dl; 6 weeks post-intervention 12.8 g/dl), very slight elevation of liver transaminases (pre-interventional GOT 27 U/l GPT 28 U/l; 8 days post-interventional GOT 104 U/l GPT 115 U/l), and a thrombocytosis (pre-interventional 279/nl; 8 days post-interventional 638/nl; 6 weeks post-intervention 376/nl) which is likely linked to the removal of about half the organ volume of the spleen. No eosinophilia was observed.

## Discussion

The presented patient resides in one of the global regions with higher prevalence of AE [1, 3]. Despite this, AE is overall uncommon and may not be suspected at initial presentation. This is especially the case in patients with a very rare primary and exclusive extra-hepatic manifestation. In our literature search on primary extrahepatic manifestations, we found three reports on spinal manifestations, three reports on splenic manifestations, two reports on skin manifestation, one report on a cerebral manifestation, and one report on a pulmonary manifestation [4–10]. Unlike in CE lesions, ultrasound is far less pathognomonic in AE and other imaging modalities play a significant role [11].

In our case, the splenic lesion was initially presumed to represent a hamartoma, a common differential diagnosis for heterogeneous splenic masses. Due to the diverse tissue types that can be present in hamartomas, these can resemble the complex, non-specific imaging presentation of AE, where solid tissue, cystic segments and calcifications can occur. When AE is considered preoperatively, guidelines recommend at least two months of pre-surgical albendazole therapy to reduce the risk of dissemination during surgery, particularly when cystic structures may rupture - as occurred in this patient. Cystic ruptures are critical for the patient outcome, as it can lead to anaphylactic reactions, and to dissemination of *E. multilocularis*. Fortunately, our patient did not suffer from an anaphylactic reaction, and up to the point of writing, there are no signs of any dissemination.

In Germany, only about 50 new cases of *E. multilocularis* infection are reported annually among 84 million inhabitants, making the disease extraordinarily rare. However, a missed or delayed diagnosis may have severe consequences for patients, such as dissemination and systemic disease. Serological testing is widely available in endemic European regions, it is a valuable diagnostic adjunct in patients with cystic lesions and is relatively inexpensive. According to a cost-effectiveness study from Austria, the serological investigation averages at €42.-. The treatment costs, however, per AE patient in the first year after initial diagnosis only, amounts to almost €19,000.- [12].

Our case highlights the importance of including AE in the differential diagnosis of cystic abdominal lesions, including those of the spleen, particularly in patients with potential exposure in endemic areas. Anamnesis regarding leisure-time activities and serologic testing can support or refute clinical suspicion. In patients with a suggestive cystic lesion and with a positive serological result, an infection with *E. multilocularis* has to be considered as probable, requiring long-term anti-parasitic treatment. The further management of patients should be conducted in specialized centres.

## Data Availability

No datasets were generated or analysed during the current study.
